# Sensor-Fusion for Smartphone Location Tracking Using Hybrid Multimodal Deep Neural Networks

**DOI:** 10.3390/s21227488

**Published:** 2021-11-11

**Authors:** Xijia Wei, Zhiqiang Wei, Valentin Radu

**Affiliations:** 1 School of Informatics, University of Edinburgh, Edinburgh EH8 9AB, UK; weizhiqiang51@gmail.com; 2 Department of Computer Science, University of Sheffield, Sheffield S1 4DP, UK; valentin.radu@sheffield.ac.uk

**Keywords:** indoor localization, sensor fusion, multimodal deep neural network, multimodal sensing, wifi fingerprinting, pedestrian dead reckoning

## Abstract

Many engineered approaches have been proposed over the years for solving the hard problem of performing indoor localization using smartphone sensors. However, specialising these solutions for difficult edge cases remains challenging. Here we propose an end-to-end hybrid multimodal deep neural network localization system, MM-Loc, relying on zero hand-engineered features, but learning automatically from data instead. This is achieved by using modality-specific neural networks to extract preliminary features from each sensing modality, which are then combined by cross-modality neural structures. We show that our choice of modality-specific neural architectures can estimate the location independently. But for better accuracy, a multimodal neural network that fuses the features of early modality-specific representations is a better proposition. Our proposed MM-Loc system is tested on cross-modality samples characterised by different sampling rate and data representation (inertial sensors, magnetic and WiFi signals), outperforming traditional approaches for location estimation. MM-Loc elegantly trains directly from data unlike conventional indoor positioning systems, which rely on human intuition.

## 1. Introduction

With a growing number of mobile applications requiring contextual information to tailor their services for user needs, location estimation is becoming crucially more important for immediate adoption. GPS is a system-level navigation method relying on satellite signals. However, indoor environments are often shielded from satellite signals. As a result, alternative methods have been proposed for performing indoor positioning, which relies on signals such as WiFi, Bluetooth, and inertial movement sensors (accelerometer, gyroscope, barometer) [1]. Such systems are heavily engineered, but this type of approach is becoming hard to adapt to edge cases and when the indoor environment changes.

Two fundamental approaches have dominated the indoor localization solutions in different forms: Pedestrian Dead Reckoning (PDR) and WiFi based Location Estimation (with the most popular version known as WiFi Fingerprinting) [2]. In PDR, the starting point is assumed to be known and using specially engineered techniques (well-defined formulations) the travelled distance and the direction of movement are estimated such that their predictions determine subsequent adjacent positions. WiFi Fingerprinting systems compare the received signal strength with pre-recorded WiFi radio maps to estimate the best matching location.

However, purposefully engineered systems fail to work when faced with unreliable sensor data (such as from accelerometers, gyroscopes and magnetometers). The drift and noise of sensor data often lead to lower location estimation accuracy. This challenge has encouraged different creative-engineered correction solutions, such as particle filters [2,3], Kalman filters, graph-conditions [4] and constraint modelling [5]. Such systems rely on mathematical formulations to make firm assumptions about the possible movement from the isolated observations of sensors. To enable such services in complex environments, the systems are tuned for each deployment setup [4,6]. Nevertheless, these hand-engineered mathematical models lose efficiency when the indoor space changes and the propagation environment is modified. Currently, no one system has reached the level of maturity and acceptance such as the GPS has for outdoor environments. This is due to the wide differences between indoor spaces and their unique characteristics. It is costly to accommodate all possible scenarios in manually configured systems and their maintenance [7].

Other solutions aim to combine the two approaches through ingenious designs (sensor fusion and independent estimation fusion). These environment-specific engineered systems make firm assumptions about the subject movement pattern based on imperfect observations from data. They often restrict the possible subject activities because irregular movements are harder to model with common mathematical formulations. Inevitably, these hand-engineered models often fail in challenging scenarios.

We advance the previous methods for performing indoor localization by adopting a convenient approach based on end-to-end multimodal deep learning. We believe that moving the focus from minutely understanding mobility patterns to learning cross-sensor patterns automatically from data is a more attractive proposition. This will allow positioning systems to be more flexible and robust due to affording to continuously update models from fresh data.

Multimodal machine learning is a proven technique for modality-fusion, such as for audio-vision speech recognition and for context recognition [8]. However, this has not been used before for multi-sensor data fusion for location tracking. We propose the first hybrid multimodal deep neural network to perform the fusion of raw sensor signals for location estimation. This is lightweight to run on modern mobile phones for tapping into their sensor signals (WiFi and inertial sensors).

The contributions of this work are as follows:

- We model the traditional methods of location estimation from sensor data with end-to-end machine learning approaches. The chosen networks avoid the need for hand-picked features. Instead, data processing models are learned automatically from data.
- We deploy a recurrent neural network—Long Short-Term Memory (LSTM) to model the sequential estimations of PDR—estimating a sequence of locations, starting from a known point and estimating the following points based on observations from sensor data.
- Performing modality fusion through a hybrid neural network—using different neural network structures on each sensing modality and fusing their representations via additional top layers. We use a recurrent neural network for the inertial sensor data processing and a fully-connected network for the WiFi modality.

## 2. Motivation and Related Work

Position estimation of smartphones inside buildings is a challenging task due to the GPS being unreliable in environments shielded by walls and ceilings. At the same time, other radio signals with longer penetration (cellular and FM) are limited to the granularity of position estimation they can offer [9]. Alternative methods have been proposed to take advantage of a broader range of sensors available on smartphones [10]. However, none have managed to produce a robust and scalable system for efficient indoor position estimation. We believe the reason is three-fold: (i) Indoor spaces are too complex to model with limited and fragmented observations about the environment (limited data), (ii) Signal distortions are inerrant in signal propagation (in light, sound, radio frequency and magnetic field, etc.), which are hard to model; and (iii) Current systems rely heavily on human designed features to be extracted from data (e.g., engineered solutions to estimate the number of steps and direction of movement).

Machine learning is a promising option due to its validated performance in several fields, including computer vision, natural language processing and pattern recognition for performing inferences from noisy data [11]. This clearly shows the advantage of automatically learning features from data and their correlations for producing a target label [12].

Using machine learning for indoor location tracking has become a popular research topic, which produces location estimation from sensor data. However, many systems are still built to operate on a single modality, which limits their performance. Given the unstable nature of signal propagation environments, modelling signals from scarce observations is relatively challenging with simplistic models. Besides, the indoor environment is formed of multiple signal modalities. Mono-modality limits the representation of the full indoor state, which in turn reduces the achievable performance of single-modality machine learning based positioning systems especially in edge cases.

We enhance artificial intelligence based indoor location tracking systems to understand the environment from different perspectives by designing a multimodal machine learning approach. This solution uses a neural network to capture in-depth features of the natural signals and to learn from their feature correlations in a sensor fusion manner. We demonstrate this on a system running with inertial sensor data and RSS data as input to produce flexible and robust location estimations.

We believe that by relying on models with high generalisation to learn directly from data, we can leverage the growing volume of data to tackle the aforementioned long-standing challenge that has limited indoor localisation. The direction forward is via a data-driven approach.

Therefore, our purpose here is to evolve our approaches from pure engineering work to an artificial intelligence based approach. This shift moves the focus from manually identifying patterns and fitting them with rigid mathematical formulations to automatically learning from data.

### 2.1. Pedestrian Dead Reckoning (PDR) on Inertial Sensors

**Figure 1:**
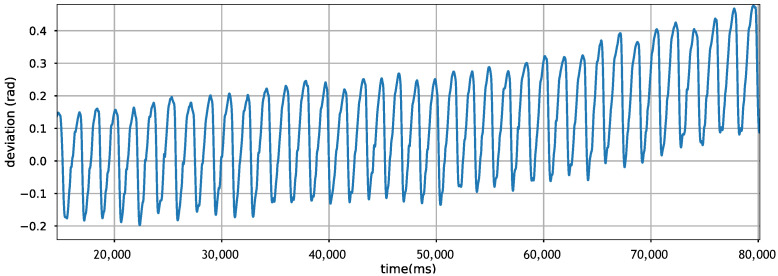
Gyroscope drifts disturbing direction calculation when sampling from a straight-line walking.

PDR builds on inertial sensors to estimate displacement distance and direction of movement. However, these sensors have their limitations. Sensor drift is one of the most notorious problems, making it hard to double integrate acceleration for displacement estimation [13]. The same problem is experienced when estimating the direction/orientation of the movement. Figure 1 shows the drift of gyroscope readings when plotting the direction of movement in a straight line. We observe that in just a few seconds, the accumulating sensor drift is substantial, changing the estimated direction of the movement dramatically. Hence, relying on the gyroscope alone is known to be inefficient, so well-engineered solutions use additional sensor inputs for recalibration [14,15].

Alternatively, to replace hard and rigid engineered solutions, others use machine learning to identify sampling characteristics in inertial sensors, such as for step size estimation [16].

### 2.2. WiFi Fingerprinting on Received Signal Strength

The WiFi Fingerprinting localization approach consists of two phases: (i) training phase or commonly known as the offline phase that collects samples to build WiFi Fingerprinting database, and (ii) the real-time phase or so-called the online phase that produces estimations based on incoming observations [2].

In terms of WiFi signal, indoor spaces experience a challenging radio propagation environment with multi-path effect, shadowing, signal fading and other forms of signal degradation and distortion. It is hard to model all the possible states of the WiFi environment in the offline phase; as such, the online phase often faces forms of the environment that was never trained on, leading to erroneous estimations.

The main challenge in taking advantage of this sensor data is that signals experienced during data collection time may be radically different to those experienced at run-time.

**Figure 2:**
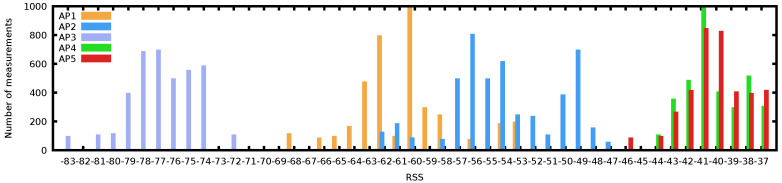
Histogram of Received Signal Strength for 5 Access Points observed at a single location showing the complexity of WiFi fingerprints, with various distributions (binomial and skewed).

Figure 2 shows the complexity of WiFi samples through the histogram of a long scan at a random indoor location. Although many WiFi-based positioning systems model RSS as a Gaussian process [17,18], we can see that none of the 5 AP histograms fits a normal distribution tightly. In fact, AP2 shows a bimodal distribution over time; AP4 and AP5 are skewed to the right and are overlapping in RSS; they are likely to interfere with each other if operating on adjacent channels. Whereas AP1 has a wide distribution of observed RSS values, spanning almost 20 dBm. Further observations on the difficulty of modelling the WiFi environment are also exposed in [4].

**Figure 3:**
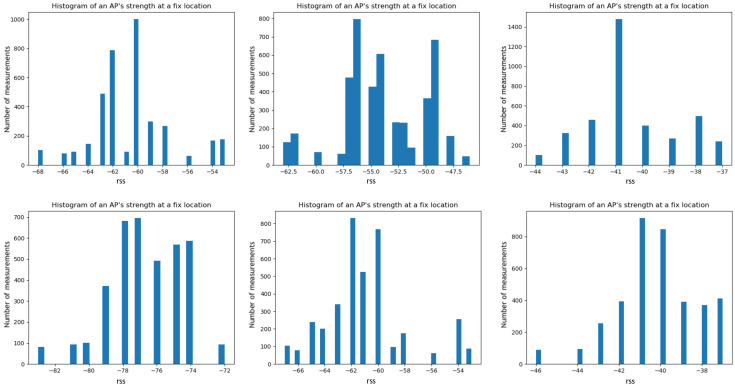
Histograms of one AP signal strength over a small time window (1 h), captured at different moments of time over a day and week. The fluctuating nature of WiFi signal, which makes this hard to model with simple function fitting.

We sampled the RSS of one AP at different fixed locations. Figure 3 shows the fluctuating nature of AP’s Received Signal Strength. Each of these histograms is drawn from one AP at a fixed location over a short period of time (within an hour). The signal is not stable across time and locations. Any slight change in the environment hinders accurate estimations. Fitting a single polynomial density function to capture this wide variation of these histograms is hard to achieve.

Unlike mathematical-based solutions, neural networks are used to tolerate these noisy data, which may be a specific characteristic of some locations, instead of simply cancelling the estimation because of noisy observations or outliers. Hence, a model should assimilate information from new data easily and capture more unexpected variations of data. Others use deep neural networks in WiFi signal strength based indoor localization [19] and also for WiFi signals with a formulation of the propagation model known as EZ [20,21], while more recent work has been using neural networks on Channel State Information (CSI) [22].

### 2.3. Multimodal Approaches

Multimodal approaches make estimations from multiple perspectives of cross-modality data possible. Filtering methods like particle filters and Kalman filters have been proposed to address the multimodality of data. Specifically, HiMLoc uses particle filters to integrate inertial sensors with WiFi fingerprints based on prior observations of Gaussian processes for direction estimation, distance estimation and correlation between samples and location in buildings and admissible human activity [2]. Similarly, WiFi-SLAM and Zee build on particle filters emphasising their importance for random system initialisation [3], while Kalman filters are used to integrate inertial sensing modalities [5]. Other engineered approaches, such as UnLoc, combine sensing modalities based on empirical observations of how some locations are unique across one or more sensors [6], MapCraft uses conditional random fields [23] and LiFS uses graph constraints to map and position estimations on the trajectory [4]. Similarly, WILL builds a connected graph to estimate location at room level [24].

Multimodal neural networks across sensing modalities have not been used before for indoor localization, although these concepts have been used before for context recognition tasks, such as human activity recognition [8]. Here, we aim to customise an end-to-end multimodal deep neural network for performing the indoor localization task such that it produces location estimation from sensor data of inertial movement samples and WiFi fingerprints. Training directly from data has its drawbacks, that of moving the challenges to the quality of training dataset (with labels) and cross-sensor modality alignment, although this can be eventually automated by other systems such as vision-based systems [25].

## 3. Methodology

In this section, we introduce the methodologies of how to use recurrent neural network for pedestrian dead reckoning based on inertial sensor data and how deep neural network performs a location estimator using WiFi fingerprints, as well as our proposed multimodal deep neural network architecture, which fuses both single-modality data to produce an end-to-end regression for estimating the coordinates of the position as output.

### 3.1. Pedestrian Dead Reckoning with Recurrent Neural Networks

PDR estimates continuous location by starting from an assumed known position and estimating displacement and direction of movement to estimate consecutive locations. By similarity, Recurrent Neural Networks (RNN) perform the same process but with the advantage of memorising previous steps and not relying entirely on new observations coming to the system, which can be affected by local noise. An RNN is an artificial neural network that includes connections between nodes that flow along a sequence. This structure is ideal for estimating time serial data.

**Figure 4:**
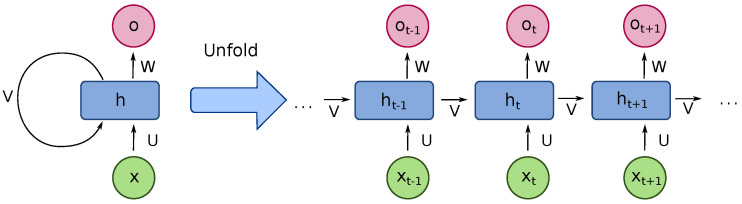
The structure of a RNN, transferring internal representations from future estimations.

RNN has proven its advantages in dealing with sequential data such as speech recognition, image captioning and machine translation tasks [26]. RNN is similar to a feedforward neural network. The difference is that the recurrent connections link a neuron from the current layer to the next neuron of the next layer. This feature makes the RNN model “remember” the features from the previous loop [27]. RNN transfers the state within each loop. Therefore, it could deal with sequential data such as the inertial sensor data we used in the experiment. We show the structure of an RNN in Figure 4.

It is an unfolded basic RNN structure that contains a number of neuron-like nodes. Those connected nodes, which are either input, output or hidden nodes, are organised into successive layers which follow a one-way direction connected to the next layer. Each neuron includes an activation that varies based on the time sequence. Errors are calculated from each sequence, while the total error is the total value of the deviations of the target label values calculated from each sequence. Practically, it operates just like the dead reckoning method, to estimate consecutive positions from streamed inertial sensor data [28].

**Figure 5:**
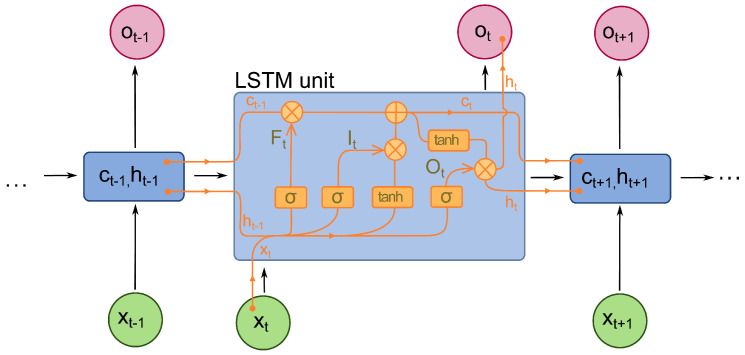
Long Short Term Memory (LSTM) Architecture.

However, the basic RNN has the problem of vanishing gradient when feeding long sequential data. It cannot catch the feature of the dependencies between samples in relatively longer sequential data. Hence, long short-term memory (LSTM) is used to solve this problem. An LSTM is an optimised RNN model that solves the basic RNN problem of vanishing gradient. This is achieved by adding a forget gate. It prevents vanishing or exploding caused by backpropagated errors. Figure 5 shows the internal structure of the LSTM unit. In each unit, there is not only an input gate and an output gate but also a forget gate that controls the “memory” to either propagate into the next layer or be forgotten in the current layer [29].

The value in the current state is controlled by the forget gate *f*. Specifically, save the value when the signal is set to 1 while forgetting the value if the gate is set to 0. The activation of receiving a new input or propagation is determined by its input gate and output gate, respectively [30]. The Equations (1) to (6) show the numerical definitions. The $x_{t}$ represents the input data at time *t* while $W_{i}$, $W_{f}$, $W_{o}$ contain, the weights of the input and recurrent connections respectively. At every time step *t*, the LSTM calculates the input ($I_{t}$), forget ($F_{t}$) and output gates ($O_{t}$) activation vectors, which decide the cell state value ($c_{t}$) and hidden cell ($h_{t}$). The softmax output (*m*${}_{t}$) determines the final probability distribution while ⊙ is the product of the cell value of the gate value.

(1)It=σ(Wixxt+Wimmt−1)(2)Ft=σ(Wfxxt+Wfmmt−1)(3)Ot=σ(Woxxt+Wommt−1)(4)ct=Ft⊙ct−1+It⊙h(Wcxxt+Wcmmt−1)(5)mt=Ot⊙ct(6)pt+1=Softmax(mt)

**Figure 6:**
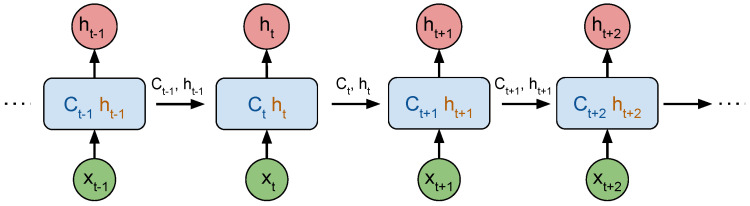
Unrolled Chain of LSTM Neural Network, using the same block for each new observation together with the internal state of previous time-step.

Figure 6 illustrates the unrolled chain of the LSTM network, where $C_{t}$ is the long-term memory at time *t* and $h_{t}$ is the block output at time *t*, or short-term memory, both transmitted to the following LSTM block in the chain.

As the sensor data is presented in time sequences, the LSTM model is ideal for location estimation. An LSTM reads the time-sequential inertial sensor data based on a fixed size of (Timestep×Features). The feature of each data point is the magnitude value of acceleration, gyroscope and magnetic field data. The number of data points in each sample is determined by the chosen time window (here, we set the time window as one second sampled every 100 ms, explained later). Each sample is offered a target position in coordinates ($X_{i}$, $Y_{i}$). The regression output of the LSTM model is the estimated position in coordinates ($X_{est}$, $Y_{est}$). The formulation for this process is presented in Equations (7) to (9).

(7)x−1=SensorData(I)(8)xt=WeSt,t∈{0…N−1}(9)pt+1=LSTM(xt),t∈{0…N−1}

During the training process, the LSTM updates its weights and state each time when reading a new incoming sensor data and returns a location estimation result. Here, $x_{-1}$ represents the inertial sensor data from the last time step; $x_{t}$ indicates the current stage of the LSTM block input, the weighted ($W_{e}$) sensor data ($S_{t}$); $p_{t+1}$ is the LSTM cell next-timestep numerical prediction of latitude and longitude coordinates based on the current stage information ($x_{t}$).

### 3.2. WiFi Fingerprinting with Deep Neural Networks

Indoor positioning systems use an anchoring mechanism to estimate the location based on instant snapshots of the environment sampled by sensors for recalibration. One such independent estimation can be achieved with WiFi Fingerprinting, which are regarded as unique at some positions [6] for localising without continuous sensing. For periodic recalibration, the WiFi is a reliable anchoring signal source, used extensively in previous research [2,3,6,24], relying on real-time observations to match the fingerprints in the database or with a pre-trained model for position estimation.

**Figure 7:**
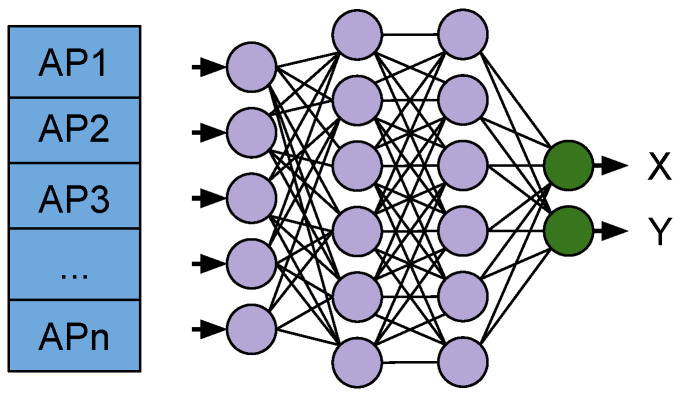
WiFi Fingerprinting DNN used for producing X, Y coordinates for the location estimation.

Here, we introduce our approach for using deep neural networks (DNN) as WiFi Fingerprinting for location estimations. A visual representation of the WiFi Fingerprinting model is presented in Figure 7.

The end-to-end DNN takes WiFi scans of observed Access Points at each sampling time-step as input and target coordinates ($X_{i}$, $Y_{i}$) for the location where the fingerprint was collected to use in the training phase. The training minimises the estimation error between the ground truth and the network estimation ($X_{est}$, $Y_{est}$). In the online phase, the network produces the estimations based on the WiFi RSS data alone.

### 3.3. Sensor Fusion via Multimodal Deep Neural Networks

By fusing both inertial sensors and WiFi fingerprints modalities, their unique perspectives can contribute to more robust estimations. Similar to our previous work in multimodal deep learning for context recognition [8], here we explore the capacity of similar construction to combine the two aforementioned neural networks operating on each sensing modality.

**Figure 8:**
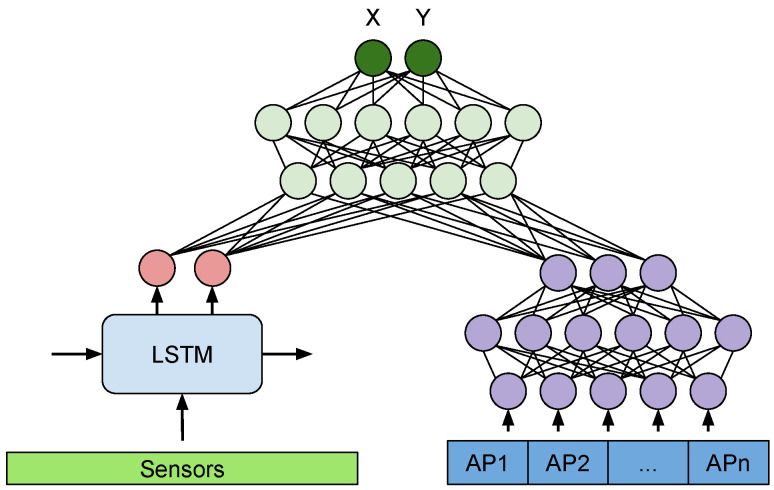
MM-Loc: our proposed multimodal deep neural network architecture for indoor localization with two parallel single-modality feature extractors and a joint network structure to merge latent features at the top.

Figure 8 presents our proposed MM-Loc architecture, an end-to-end multimodal deep neural network for performing indoor localization, which makes inferences on the joint perspective of inertial sensors and WiFi fingerprints modalities.

The MM-Loc takes time-sequential inertial sensor data through the LSTM sub-network and WiFi RSS data through the DNN sub-network (when available) in parallel. The input size of the sensor modality at the LSTM side is (Timestep×Sensor_num) and for the DNN side matches the WiFi Fingerprint vector (AP_num). Both modalities are reduced to 128-dimensional hidden units (internal representation) on each branch. These two parallel 128 units are then fused by concatenation to 256 units. The 256-dimensional new representation is fed into three fully connected (FC) layers with 128 and 64 hidden units, before the final output layer at the top performing a two-dimensional regression ($X_{est}$, $Y_{est}$).

The inference process is done in one pass through the MM-Loc network, taking the high-frequency inertial sensors at each estimation step, while the WiFi fingerprint input vector is provided on the DNN branch when available. If a WiFi sample is not collected (due to its lower sampling frequency or because it is missing for various reasons), a null vector is provided as input for the WiFi branch, leaving the estimation on the LSTM side alone. In training, the gradient flows through both branches, updating the weights if they had any contribution. A null vector as input on the WiFi branch causes neglectable changes to that branch during training.

What is unique about this construction is its ability to handle imbalanced sampling rates or missing samples from the WiFi modality. This is because WiFi scans are produced at a much lower rate than inertial sensors, so when there is no WiFi scan in the system, the WiFi input is a vector with all components value of 0 (normalised to −100 dBm). This null vector causes the inference to balance entirely on the inertial sensors side. When both modalities have inputs our multimodal architecture combines their perspectives and trains both branches.

## 4. Data

We collect a multimodal sensor dataset from two scenarios with ground truth location labels. Our preprocessing involved granular location interpolation, normalisation, overlapping and downsampling.

### 4.1. Data Collection

**Figure 9:**
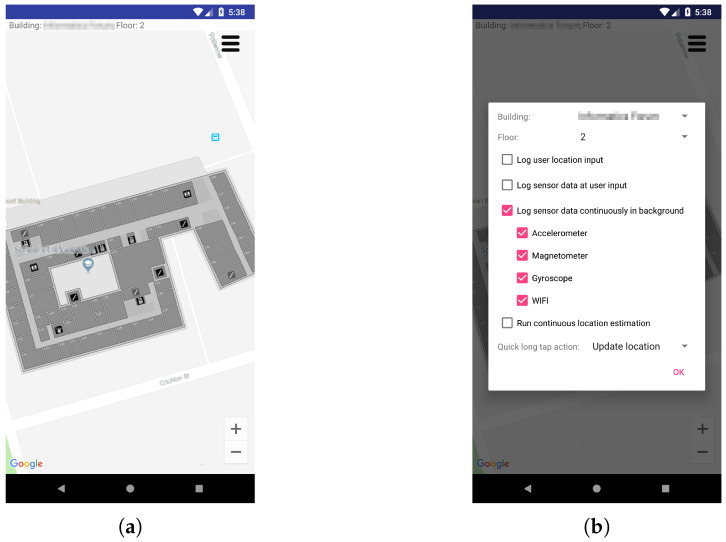
Screenshots of the Android application used to collect multisensory data. (**a**) Location Input Interface; (**b**) Sensors Control Options.

The multimodal sensor dataset is collected using an Android application designed specifically for the task of data collection. Figure 9 presents screenshots of the mobile app graphical interface for initiating collection and labelling of sensor data. This application can be configured to collect sensors data (accelerometer, magnetometer, gyroscope and WiFi scans) continuously in the foreground with a visual interface to accept user inputs or running in the background, to allow continuous data collection with phone in pocket and screen switched off. To collect data with ground truth location labels, we run the same application synchronously on a second mobile phone. One marks locations as input through the visual interface displaying the building map aligned to Google Maps coordinates for the purpose of longitude and latitude information acquisition. The logged ground truth geographical locations are converted to the Universal Transverse Mercator (UTM) in metres for our experiments. The second phone is carried by the user to collect sensor data continuously running our application in the background. There is no user interaction while collecting data to resemble the perspective of sensors in natural motion.

During the data gathering process, ground truth labels are transferred between the two devices. The first data collection campaign involved walking on the corridor at different walking speeds, starting from one corner of the building, performing a circular trajectory and arriving back at the starting point. Ground truth locations were provided sporadically, but to obtain location information on a more granular basis, some locations were interpolated between consecutive two input locations—assuming local constant walking speed. Specifically, during one round of data collection, we build two record sets synchronously: Set.1 holding the inertial sensors and WiFi scan from walking on the corridor with phone 1; Set.2 the ground truth locations as labels collected with phone 2 by inputting the latitude and longitude information of phone 1, when passing key locations such as corners and doors.

For training our MDNN model, we use two scenarios into a training set—collected from two crowded office buildings. Both scenarios are typical indoor environments with diverse human activities that increase the complexity in terms of noisy data and variation. Specifically, these complex situations include people walking alone the person who collected the data, which affects the normal traversal of corridors during data collection; various electronics in operation, such as elevators, computers, printers and portable devices, which generate electromagnetic interference; building materials contain reinforced concrete, metal and glass, which influence signal propagation patterns. Furthermore, we use multiple mobile devices with different built-in sensor sensitivity and sampling rates for data gathering, which adds to the complexity of the experiment. During data collection, the participants walked at different speeds and were free to exercise other postures which again adds to the motion complexity.

Table 1 presents the samples distribution of the two data collection scenarios. Scenario A holds 24,450 inertial sensor samples and boosted number of WiFi samples, to 25,541, by interpolating with static samples at precise locations. Scenario B holds fewer WiFi samples, 8390, but collected at the same time with the inertial sensors, 29,836 samples.

### 4.2. Data Preprocessing

We pre-process our data to match a standard format for training and testing. This is challenging because of the imbalanced sampling rate of different sensors.

#### 4.2.1. Inertial Sensor Data

The Android API provides sensor samples on an event base, only if the value is considered to have changed. This leads to uneven intervals between sensor samples. Inertial sensors—accelerator, gyroscope and magnetometer—hold different refreshing rates. We use linear interpolation to fill the missing values in between every two hardware sensed values in order to generate the continuous time-sequential data. The interpolated data is grouped in time windows, which we discussed later, and has a location associated to it. We interpolate geographical locations based on the selected time window between each two recorded locations as we only record locations when passing special points such as corners, elevators, kitchen, meeting rooms and other obvious landmarks along the trajectory.

The inertial sensors sample the motion in three orthogonal directions, on axes Ox, Oy and Oz. Because the phone can be placed in any orientation in a pocket or in a bag, invariant sample data is attained by calculating the magnitude value of the built-in sensor (accelerometer, gyroscope and magnetometer) from the values on the three axes (Ox,Oy,Oz), as:

(10) $${\mathrm{sensor}}_{magnitude}=\sqrt{{\mathrm{sensor}}_{x}^{2}+{\mathrm{sensor}}_{y}^{2}+{\mathrm{sensor}}_{z}^{2}}$$

As the LSTM load the time-sequential data by time window, a proper window selection contributes to lower computational cost and on-device power consumption with acceptable inference efficiency. We explored four time-window sizes: 10 ms, 100 ms, 1000 ms and 2000 ms. In addition, we considered the case of time windows overlapping (10%, 50%, 90%) to increase the frequency of location updates for a fast responsive system. Window overlapping also plays well with the LSTM model since the information from previous time windows is reinforced and emphasised by overlapping for better quality detection and strengthening of correlations between samples.

**Figure 10:**
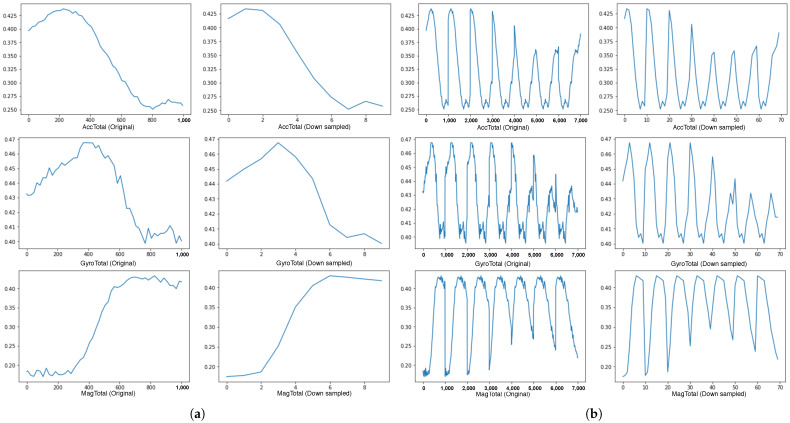
Compare between the original data and the downsampled data. It shows that loss of information is minimal across two time windows, being able to follow the trend in signal for the walking activity. (**a**) Sensor values over a time window of 1 s; (**b**) Sensor values over a wider time window of 7 s.

Despite the amount of machine learning dataset increased with richer information for training by time window grouping and overlapping, the side effect is that the heavier dataset increases the processing demand. Hence, to improve the feed-forward speed with a smaller input size without losing too much information, a downsampling method is explored to compress the dataset. Figure 10a shows three magnitude values of the accelerometer, gyroscope and magnetometer with 1000 data points (1000 ms) on the left side and the downsampled value to as much as 90% linear compression. Figure 10b compares the original data and the downsampled over a longer interval of 7000 ms. The comparison from Figure 10 indicates that the main features are maintained even on high-level compression, reducing the frequency of samples to 1 Hz. As such, we analyse the downsampling effect in the experiments presented in the evaluation section.

#### 4.2.2. WiFi Fingerprint Data

The RSS inputs to the neural networks are provided as vectors. To construct this vector, we first scan the whole WiFi logfile to identify all unique APs observed throughout the data gathering process (total of *n* APs observed inside the building), as well as the minimal and maximum received signal strength encountered throughout. These values are used to normalise the vector input to the [0, 1] interval by linear scaling. We observe the min-max interval is [−99, −40] in dBm. Hence, for missing APs in WiFi scans, a value of −100 is associated with their position in the n-dimensional vectors. To produce a WiFi fingerprint vector as realistic as possible, we keep even the occasional personal hotspots found throughout the data gathering. These APs act as noise in the dataset, to increase its complexity.

### 4.3. Sensor Fusion Dataset Alignment

The main challenge of aligning inertial sensor data with WiFi data is that the WiFi sampling frequency is significantly lower than the inertial sensors sampling rate due to hardware characteristics.

By observing the original sampling rate of sensors from the log files, we determine the average sampling frequency of the inertial sensor to be in milliseconds, while the WiFi updates come in seconds apart. (The time gap between WiFi samples is a non-integer value, such as 1.03 s, 3.56 s, etc.). The amount of time-series sensor data is significantly larger than WiFi samples.

If we simply combine these two modalities (one-second grouped inertial sensor data with one WiFi scanning as one input data point), the set contains sparsity with unbalanced data components. This is because the WiFi scans are many times less frequent than one second as we discussed. For the purpose of eliminating the sparsity of the WiFi data and to increase the location estimation frequency, we adjust the WiFi scan rate from the original sampling rate to every 100 ms. For instance, if the first time duration of 0.1 s in the log file contains three WiFi scans, we compress these three samples into one single sample. Therefore, we get a denser WiFi fingerprint dataset to be aligned with a time window of inertial sensor data, illustrated in Table 2.

If the time window contains only inertial sensor samples, with no WiFi scan, we use −100 dBm to represent missing APs in an abstract WiFi scan, to indicate that a scan was not available for that time window.

As two synchronously-logged datasets contain not only inertial sensor and WiFi RSS samples but also ground truth location information within the same time duration, the timestep records are utilised for matching multimodalities with geographical labels. The processing of locations involves normalising the coordinates to the bounding boxes chosen for the building and scaling to the interval [0, 1]. Estimations of neural network models are converted back into latitude and longitude coordinates. Table 3 indicates the generic elements of the records used for training after the time alignment of sensor samples.

## 5. Model Configuration

This section presents the evaluation of each independent modality-specific neural network architecture, followed by the evaluation of the multimodal deep neural network (MDNN) implementations with different fusion architectures that combine the features extracted by the two independent models (RNN for sensor data and DNN for WiFi samples) to produce a new location estimation at the top.

### 5.1. Recurrent Neural Network on Inertial Sensors

Here we present our exploration to identify the best model structure and parameters for calibrating the LSTM for the best performance in terms of time window settings, overlapping ratio and data compression.

#### 5.1.1. Time Window

As LSTMs read the data in time windows, a well-selected time window allows the model to catch enough detailed information needed for estimating the movement accurately. A larger time window loses granular information by exploiting larger scale observations, including more information for a range of movements while being computationally demanding for performing inferences on mobile devices and slower in providing location updates. In contrast, a smaller time window captures minimal information, not discriminating between different walking speeds or between very similar activities like moving on a flat surface and climbing stairs, although more computationally friendly to mobile devices since the input layer is smaller. We train the LSTM model with the time window of 10 ms, 100 ms, 1 s and 2 s, and the model hyper-parameters listed in Table 4.

**Figure 11:**
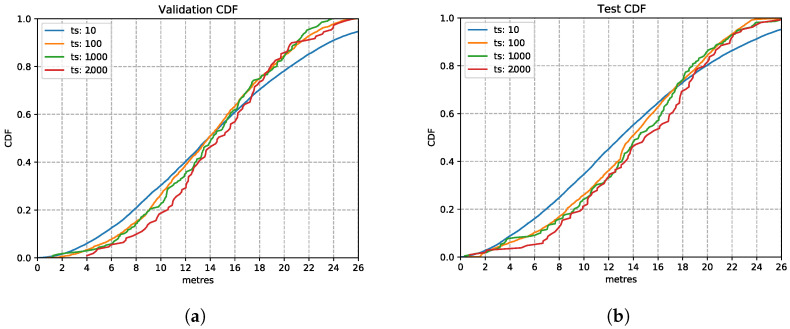
LSTM model performances with different time window settings. (**a**) Validation Set CDF. (**b**) Test Set CDF.

Figure 11 shows the LSTM performance with various input time windows, presented using the Cumulative Distribution Function (CDF) charts. As observed here, the time window setting has limited impact on the inference accuracy. This observation on the validation set is also confirmed on the test set in the CDF plot Figure 11b, which shows that several time windows have a similar location estimation accuracy. The 1000 ms based model shows a good performance similar to the others and going for this larger time window allows the model to capture more observations. The estimation error is still high since this is the first parameter we optimise for. Therefore, we choose the time window setting of 1000 ms in the following explorations.

#### 5.1.2. Overlapping Ratio

This evaluation presents the outcome of changing the overlapping ratio based on the fixed time window setting of 1000 ms. The overlapping ratios we experiment with are 30%, 50% and 90%, which increase the amount of training data subsequently by 1.3×, 2× and 9× respectively.

There are two main advantages to implementing overlapping. The first one is to enhance dependency between consecutive time window samples by repeating information observed in the overlapping part. With the LSTM model, this enhances the memory aspect of adjacent time windows, the model experiencing portions of the recent action over consecutive inputs. Moreover, we increase the training dataset synthetically by obtaining a larger number of training samples, since larger training sets help with the training of neural networks.

**Figure 12:**
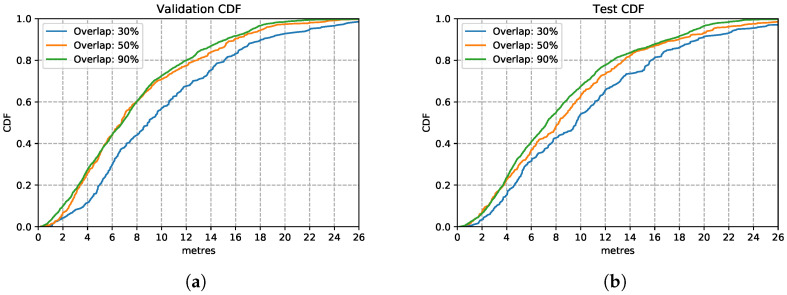
LSTM model performance with different overlapping rations on validation and test set. (**a**) Validation Set CDF. (**b**) Test Set CDF.

Figure 12 shows the three models trained with the enhanced training sets after introducing overlapping of samples. In the training set, it appears that the model trained with data overlapping by 90% performs consistently better than the other two models trained on 30% and 50% overlapping data. This is also the case over the test set, in Figure 12b, the model trained with overlapping 90% of samples performs similarly well as in the training set. This is a good performance, considering that it is based on nothing more than inertial sensor data without much to calibrate on apart from occasional well-located changes of direction (e.g., when going around a corner). This route covered long stretches of straight-line corridors (up to 60 m) walked at various speeds. These conditions are recognised well by the LSTM estimator. These results illustrate that by increasing the overlapping rate, models can learn features better since they have more data available to train on.

From this experiment of using overlapping strategies, model performance improves compared to those without time windows overlapping. The 90% windows overlapping model has the best performance. We use this training enhancement in the following experiments.

#### 5.1.3. Data Compression

We implement simple downsampling and Principal Component Analysis (PCA) strategies for data compression to reduce the cost of training and inference time. As mentioned in Figure 10, simply downsampling has little impact on the original signal representations. Hence, we implement downsampling (pick up datapoint every 100 ms) over the training data with the overlapping ratio of 90%. Therefore, one sample has been downsampled from the size of (1000×3) to (10×3). For the PCA data compression, the dimension is reduced to the same size of (10×3) for each sample. New variables in a lower dimension could be calculated based on eigenvalues and eigenvectors and therefore replace the original variables in a higher dimension matrix by PCA.

**Figure 13:**
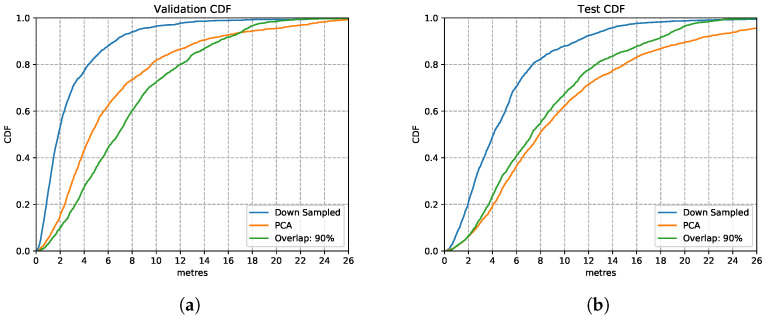
LSTM Model performances with different data compression strategies. (**a**) Validation Set CDF. (**b**) Test Set CDF.

Figure 13 shows the comparison between the downsampled based model and PCA based model with an overlapping ratio of 90% for the LSTM model. Both CDFs illustrate that the downsampled based model performs better than the uncompressed data based model in that it reaches 80% of the prediction accuracy with the precision requirement of 8 m on the test set. PCA based model performs slightly better compared to the uncompressed model in both validation and test set.

In general, the downsampled based model has the highest accuracy and reliability compared to the PCA-based and to the model without data compression. By implementing downsampling to the overlapping data, the model reduces its complexity by setting a smaller time step to save computation time. It could handle the data with a larger time window without losing significant data features. Specifically, ten datapoints in one time window represent the data features of one-thousand samples in a time window, which allows the model to further increase the time window allowance with little model complexity increase. It could potentially allow the model to process a variety of activities on short input vectors. This is of critical importance when integrating the model into mobile devices to improve the prediction efficiency, which reduces application response time and power consumption.

### 5.2. Overview of RNN for Inertial Sensors

**Figure 14:**
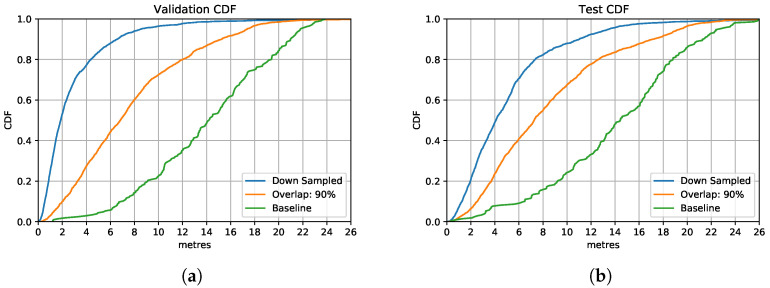
Overall comparison of model performances with different calibration strategies. (**a**) Overall Validation Set CDF. (**b**) Overall Test Set CDF.

Figure 14 contains all models discussed in this section. Overall, starting with selecting a suitable time window from 10 ms, 100 ms, 1 s and 2 s, we choose 1 s for the time window size, as this allows more data variation. We then improve estimation accuracy by further increasing the training size with overlapping. For 90% overlapping of sensor samples, the model shows a significant improvement in location estimation accuracy. To reduce the complexity and the training time of the models, we compress the training data size by downsampling and PCA dimension reduction. We selected the LSTM model with downsampling based on a 90% overlapping ratio, presented by the blue line in Figure 14, as the inertial sensor positioning model settings, which balances the estimation accuracy and efficiency.

### 5.3. Deep Neural Network on WiFi Fingerprints

WiFi scans are received at an average update rate of about one second on experiment smartphone devices of Samsung Galaxy S6 and HUAWEI P40. We use a Deep Neural Network (DNN) as the WiFi Fingerprinting model, which takes WiFi scans from sensed Access Points (AP) Received Signal Strength (RSS) at each sampling timestep, as input to produce estimations, the normalised latitude and longitude. We evaluate the WiFi estimator regarding model structure settings on the WiFi fingerprints dataset. The missing APs are represented by −100 dBm and converted to zero when normalising to the [0, 1] interval for input.

#### 5.3.1. Model Structure

**Figure 15:**
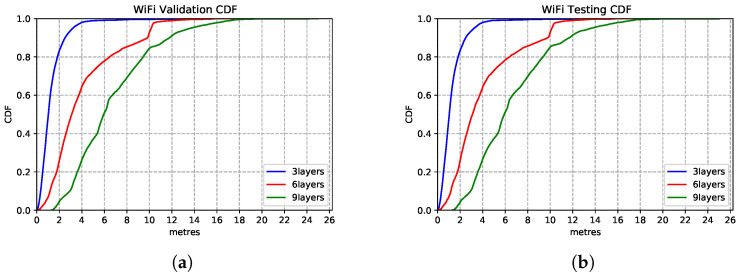
WiFi based DNN model performances with different network structures. (**a**) Validation Set CDF. (**b**) Test Set CDF.

We explore the impact of architecture settings of 3-layer, 6-layer and 9-layer DNN structures. As we observe from the CDF in Figure 15, the 3-layer DNN regression model produces the best inference accuracy compared to the deeper networks. The models show extreme similar estimation capability on the test set—Figure 15b, which shows the model generalisation.

#### 5.3.2. Model Tuning

After we determine the DNN structure, we implement model tuning on the 3-layer WiFi DNN regression model with the hyperparameter settings shown in Table 5. It should be mentioned that the only variation of the model structure is the input sizes caused by the number of APs sampled in different experiment buildings. For our two evaluation scenarios, there are 102 APs and 750 APs, respectively.

### 5.4. Overview of DNN for WiFi Fingerprints

The WiFi-based estimator is modelled with a deep neural network regression model. By exploring the model structure with parameter tuning settings, we decide to use a three-layer DNN model for WiFi Fingerprinting. We use this WiFi model architecture as the sub-component network integrated into the multimodal fusion model.

### 5.5. Multimodal Deep Neural Networks on Sensor Fusion

After we determined the model structure for each modality, we move our focus to fuse each modality-specific component into a uniform Multimodal Deep Neural Network (MDNN).

#### 5.5.1. MDNN Integration

To explore the best fusion architecture of the MDNN that links the modality-specific features extracted from the RNN and DNN sub-networks as introduced in Figure 8, we customise four types of fusion networks, including two hybrid element-wise fusion of concatenation and multiplication, a hybrid residual connection fusion as well as a late fusion structure.

**Element-wise Fusion:** The MDNN with element-wise fusion architecture is shown in Table 6. By concatenating the modality-specific hidden layer outputs from both LSTM and DNN sub-networks of 128 dimension output, the fusion layer read these two hidden outputs by implementing element-wise matrix calculation of concatenation (128×2) or multiplication (128). This fused matrix then goes through higher 128 and 64 size fully-connected joint layers and eventually are regressed to a two-value prediction ($X_{est}$, $Y_{est}$).

**Residual Connection Fusion:**Table 7 shows the MDNN with a residual connection architecture. Different from the element-wise fusion MDNN, in order to emphasise the WiFi features which are smaller in representation compared to the time-sequential inertial sensor data, we add a residual connection layer that transfers the hidden output (128) from WiFi penultimate fully-connected layer to the joint layer, fusing together with the LSTM (128) and DNN last FC layer outputs (128×2). This derives a 128×3 representation for the sensor-fusion component, which performs the final location estimation.

**Late Fusion:**Table 8 presents the MDNN architecture with the late fusion strategy. This works by combining two separate LSTM and DNN model outputs, the predictions that produce the lat-long coordinate estimation (*X*_Sensor_, *Y*_Sensor_) and (*X*_WiFi_, *Y*_WiFi_) respectively. These estimations form a four-dimensional feature input vector, which provides the representations needed by the top layers to estimate the final latitude and longitude (*X*_Fusion_, *Y*_Fusion_).

#### 5.5.2. MDNN Implementation

**Figure 16:**
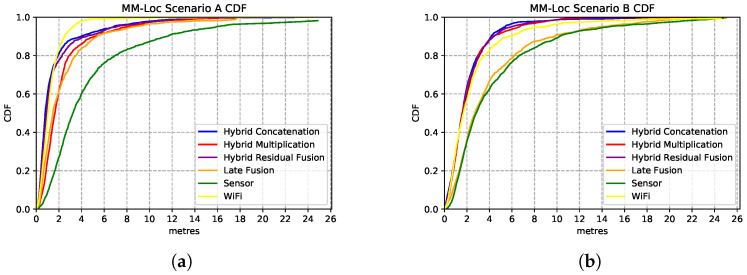
Comparison of MDNN model performances with different fusion architectures. (**a**) Comparison CDF on Scenario A; (**b**) Comparison CDF on Scenario B.

We present the estimation accuracy from the four MDNN with different fusion strategies, comparing sensor and WiFi single-modality location estimators results. The CDF charts in Figure 16 show the estimation strength of these four models. We evaluate these models on the aligned multimodal dataset collected from two buildings (deployment scenarios) with the following split radio: 65%, 25% and 10% for training, validation and testing, respectively.

Figure 16a shows the performances of the MDNN with hybrid concatenation, hybrid multiplication, residual connection and late fusion architectures, as well as the sensor model and WiFi model on scenario A. We observe that the hybrid concatenation fusion MDNN performs the best with 1.98 m precision in 80% of the estimations, followed by the residual fusion and multiplication fusion models. Although the late fusion model has a relatively poor 3.7 m accuracy, it is approximately 2× better than that from the sensor-based estimator. In terms of single-modality estimator performance, the WiFi model performs significantly better than the sensor models with 2.6× better accuracy. In 80% of the estimations, the WiFi model has an accuracy of 2.6 m error while the sensor model holds 6.9 m prediction error.

We observe similar performances from these models on Scenario B (second building), with the best performance contributed by the same architecture of concatenation fusion MDNN, just under 2 m median error, as shown in Figure 16b. Regarding single-modality model performances, the WiFi model has an error of 3 m, and the sensor-based model has an error of 6.2 m.

### 5.6. Overview of MDNN on Sensor Fusion

By evaluating the performance of MDNN under different fusion architectures (concatenation, multiplication, residual connection and late fusion), we observe that the median error of the hybrid fusion multimodal model on the test set is 1.98 m, which is significantly lower than other fusion models and modality-specific estimators from sensors and WiFi data. As observed from the CDF plot in Figure 16, 90% of the errors are lower than 4 m in both scenarios. The hybrid concatenation MDNN fusion method has the best estimation accuracy among all other models under both scenarios, which gives us confidence in the generalisation power of the models. We use the element-wise concatenation as the fusion method for the MDNN, giving it the name of MM-Loc, for further evaluations.

## 6. Evaluation

In this section, we present the results of evaluating the MM-Loc performance under both scenarios, compared to modality-specific models performances. We also discuss the inference accuracy influenced by sensor inputs with different sampling frequencies. Furthermore, we add a comparative study for evaluating our proposed MM-Loc with other state-of-the-art (SOTA) multimodal positioning systems.

**Figure 17:**
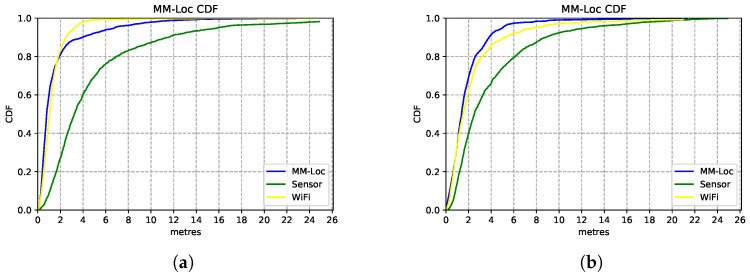
MM-Loc prediction CDF compared with modality-specific models. (**a**) MM-Loc Performance CDF on Scenario A; (**b**) MM-Loc Performance CDF on Scenario B.

MM-Loc’s median accuracy is within 2 m error for 80% of the prediction cases, which is 3.5× better than the single-modality baseline model. By comparing Figure 17a,b, we observe that in scenario A the MM-Loc performs better than the WiFi-based single-modality model within 2 m error at the intersection point with the WiFi model. However, the WiFi model outperforms the MM-Loc for larger precision tolerance. In scenario B, MM-Loc always outperforms the single-modality models. This is likely due to the WiFi signal coverage being denser in scenario A than that in scenario B, which contributes to the WiFi-based position estimator quality. As observed, the MM-Loc outperforms single-modality models.

To explore the opportunity for reducing energy consumption, we vary the WiFi scan frequency in our proposed MM-Loc system. Specifically, for scenario A, as the default WiFi sampling rate is 10 Hz, sourced from the system, we reduce the scanning frequency of the dataset from 10 Hz to 5 Hz and 1 Hz by applying a filter. The purpose of adjusting the WiFi sample frequency is to assess the impact of this energy-saving strategy of scanning reduction onto the location estimation accuracy. This also shows how our model behaves in systems where a high refresh rate is not available. In Scenario B, we decrease the WiFi sampling frequency from the original 1 Hz to 0.1 Hz and even 0.05 Hz for the same reason.

**Figure 18:**
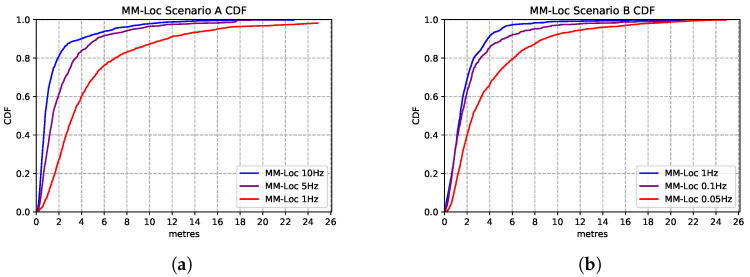
Comparison of MM-Loc model performances running at different WiFi sampling rates. (**a**) MM-Loc Performance CDF on Scenario A. (**b**) MM-Loc Performance CDF on Scenario B.

Figure 18 presents the comparison between the performances of MM-Loc running at different WiFi sampling frequencies. We found that the multimodal model prediction accuracy experiences the same trend with the decrease of sampling rates. MM-Loc with an intermediate sampling rate still predicts with approximately 4 m accuracy. Hence, the sampling rate has little impact on accuracy, but it can bring a lot of savings on-device from reduced computations and WiFi scans.

**Figure 19:**
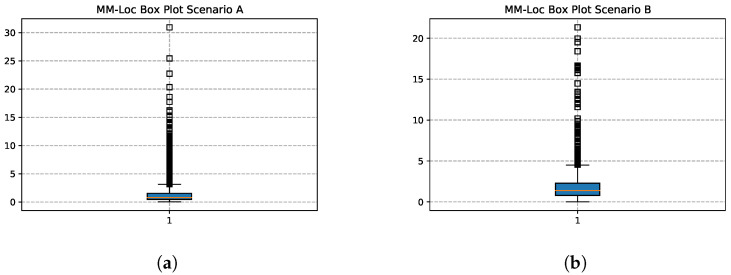
MM-Loc Prediction Box Plot. (**a**) MM-Loc Box Plot on Scenario A; (**b**) MM-Loc Box Plot on Scenario B.

Figure 19 shows the box plots of the MM-Loc with the default WiFi frequency inputs in both scenarios. In scenario A, the location estimation accuracy at first quartile ($Q_{1}$), second quartile ($Q_{2}$) and third quartile ($Q_{3}$) is 0.467 m, 0.784 m and 1.545 m respectively; while in scenario B, $Q_{1}$, $Q_{2}$ and $Q_{3}$ is 0.789 m, 1.387 m and 2.288 m respectively.

### 6.1. MM-Loc Comparative Study

We compare our proposed MM-Loc with other multimodal SOTA positioning systems on the same dataset collected from the two scenarios. These SOTAs include P-MIMO, which uses RNN to extract multiple received signal strength and DNN for predicting regression location outputs [31]; HDLM, which uses a convolutional neural network (CNN) for RSS feature extractor and LSTM for regression locations estimations [32]; GRU-CNN, which uses CNN to extract RSS features and Gated Recurrent Unit (GRU) for positioning predictions [33]. The estimation performances are shown in Table 9. Compared with other SOTA baseline models, our proposed MM-Loc has the best performance, which shows the superior position estimation capability with the highest prediction accuracy and the lowest estimation error in both scenarios.

**Figure 20:**
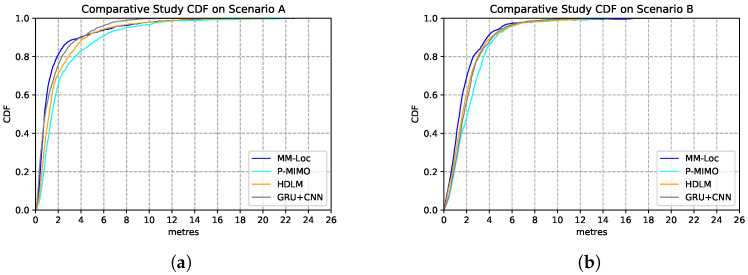
Comparison of model performance CDF of MM-Loc and SOTA models. (**a**) SOTA Performance CDF on Scenario A; (**b**) SOTA Performance CDF on Scenario B.

Figure 20 represents the CDF performances of the MM-Loc model and the aforementioned SOTAs models. As it can be observed, in more than 90% of the estimations, our MM-Loc outperforms all other SOTA estimators.

### 6.2. MM-Loc Visualisation

**Figure 21:**
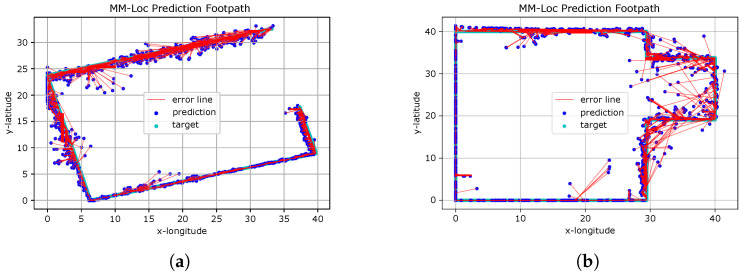
MM-Loc Footpath Visualisation. (**a**) MM-Loc Predicted Footpath on Scenario A; (**b**) MM-Loc Predicted Footpath on Scenario B.

Figure 21 presents the predicted footpath from the MM-Loc for two scenarios. The red line indicates the distance between the coordinates of the ground truth and estimated locations produced by the MM-Loc model.

We observe that MM-Loc predicts the footpath along the corridor with high fidelity, having clear estimation boundaries. However, some predictions are over 5 m away from the ground truth, especially at the corners of corridors. This is likely an effect of the difficulty of observations in the WiFi component near corners. The other aspect introducing errors is the magnetic interference present in some places on the pathway (elevators and heavy iron materials in building materials).

## 7. Discussion

Our work shows that conventional smartphone indoor localization methods can be modelled by end-to-end deep neural networks and fused by a multimodal structure. This starts with individual feature extractors specific for each modality (using RNNs and DNNs) and then fusing these representations for the final inference through joint neural network architecture. With this, we are moving the effort from engineering each modality component (step counting, direction estimation) and other conventional integration methods (particle filters, Kalman filter and graph-based constraints) to a purely data-driven machine learning effort.

Our data-driven fusion approach builds entirely on the quality and volume of data, without engineering preliminary features nor making assumptions about the use of the system. Previous systems fail when porting to new environments because of the built-in assumptions about the scene. In contrast, our system is generalizable as it requires low deployment costs. This is because the MM-Loc model can be automatically retrained to the new deployment scenarios by transfer learning. The training relies on data samples with minimum effort for data collection and labelling.

The proposed fusion approach of our system is extendable to include other various sensing modalities and signal sources (such as light, environmental noise, humidity, air pressure, etc.) for improving the system performance.

There is still space for improvements in the estimation accuracy. Despite our exploration with a low volume of training data, we show that even these limited training sets are enough for our end-to-end machine learning multimodal DNN solution to produce good estimations. Our method moves the effort entirely on the quality of the training data. Although data collection is still a hard challenge for now, we believe this is the only way to capture the fine details that are commonly missed by traditional modelling approaches. This information will always be available to train on if larger volumes of data become available. In the future, this data collection can be automated, by robots roaming the indoor space to update WiFi radio maps or by mass unlabelled data collection from users roaming naturally in the environment, as well as through labelling solutions based on computer vision [25]. The performance of our system improves with more training data becoming available.

## 8. Conclusions

In this work, we introduce an end-to-end machine learning system, MM-Loc, which uses a hybrid multimodal deep neural network to perform the task of smartphone indoor localization. Our MM-Loc is an entirely data-driven approach. We model the conventional methods of indoor localization, WiFi Fingerprinting and Dead Reckoning through neural network structures. These are capable of performing location estimation independently, with a median error of 2.8 m by the WiFi Fingerprinting neural network and a median error of 6.5 m by the inertial sensors recurrent neural network, respectively. To perform the fusion, we developed MM-Loc as a multimodal structure that bridges internal representations from modality-specific networks into a more robust location estimation solution. Our MM-Loc achieves a performance of 1.9 m median error, while being easy to deploy due to learning only from data automatically.

## Figures and Tables

**Table 1:** MDNN Dataset Description.

Datasets	Inertial Samples	WiFi Samples	Access Points	Time Duration
Scenario A	24,450	25,541	102	407 Mins
Scenario B	29,836	8390	750	497 Mins

**Table 2:** The raw WiFi Fingerprint data format. Missing APs from the current scan are indicated with Null. Each WiFi scan has an assigned collection location (X, Y) as label.

Time	AP${}_{0}$	AP${}_{1}$	…	AP${}_{n}$	X	Y
$t_{0}$	Null	−85	…	Null	$x_{0}$	$y_{0}$
$t_{1}$	−92	Null	…	Null	$x_{1}$	$y_{1}$
$t_{2}$	Null	Null	…	Null	$x_{2}$	$y_{2}$
$T_{\prime }$	−92	−85	…	Null	$X_{\prime }$	$Y_{\prime }$

**Table 3:** Cross-sensor data format, showing the normalised and filtered/interpolated values. A one second time window holds 1000 samples from each sensor, a WiFi scan and the ground-truth location. Missing APs in the WiFi scan are indicated with a −100 value.

Time	Accelerator	Gyroscope	Magnetometer	AP${}_{0}$	AP${}_{1}$	…	AP${}_{n}$	X	Y
$T_{0}$	$a_{0}$∼$a_{999}$	$g_{0}$∼$g_{999}$	$m_{0}$∼$m_{999}$	−100	−85	…	−100	$X_{0}$	$Y_{0}$
$T_{1}$	$a_{999}$∼$a_{1999}$	$g_{999}$∼$g_{1999}$	$m_{999}$∼$m_{1999}$	−100	−100	…	−100	$X_{1}$	$Y_{1}$
$T_{2}$	$a_{1999}$∼$a_{2999}$	$g_{199}$∼$g_{2999}$	$m_{1999}$∼$m_{2999}$	−70	−100	…	−65	$X_{2}$	$Y_{2}$
…	…	…	…	…	…	…	…	…	…
$T_{n}$	$a_{n}$∼$a_{n+999}$	$g_{n}$∼$g_{n+999}$	$m_{n}$∼$m_{n+999}$	−100	−100	…	−100	$X_{n}$	$Y_{n}$

**Table 4:** LSTM Model Parameter Settings.

Parameter	Settings
Epoch	100
Batch Size	100
Hidden Units	128
LSTM Layer	1 Layer
Learning Rate	0.005
Learning Rules	RMSprop

**Table 5:** WiFi-based DNN Parameter Settings.

Parameter	Settings
Input Size	AP Number
Epoch	100
Batch Size	100
Hidden Units	128
DNN Layer	3 Layers
Dropout Rate	0.5
Learning Rate	0.001
Learning Rules	RMSprop

**Table 6:** MDNN Architecture with Element-wise Fusions.

Layers		Output Shape	
LSTM Layer (sensor)		(Batch Size, 128)	
FC Layer.1 (WiFi)		(Batch Size, 128)	
Dropout Layer.1 (WiFi)		(Batch Size, 128)	
FC Layer.2 (WiFi)		(Batch Size, 128)	
Dropout Layer.2 (WiFi)		(Batch Size, 128)	
FC Layer.3 (WiFi)		(Batch Size, 128)	
Fusion Layer (concat or multiply)		(Batch Size, 128 × 2 or 128 × 1)	
FC Layer.4 (joint)		(Batch Size, 128)	
FC Layer.5 (joint)		(Batch Size, 64)	
FC Layer.6 (joint)		(Batch Size, 2)	
**Batch Size**	**Learning Rate**	**Learning Rules**	**Dropout Rate**
100	0.001	RMSprop	0.5

**Table 7:** MDNN Architecture with Residual Connection Fusion.

Layers		Output Shape	
LSTM Layer (sensor)		(Batch Size, 128)	
FC Layer.1 (WiFi)		(Batch Size, 128)	
Dropout Layer.1 (WiFi)		(Batch Size, 128)	
FC Layer.2 (WiFi)		(Batch Size, 128)	
Dropout Layer.2 (WiFi)		(Batch Size, 128)	
FC Layer.3 (WiFi)		(Batch Size, 128)	
Residual Layer (FC Layer.2 WiFi )		(Batch Size, 128)	
Fusion Layer (LSTM, FC Layer.3, RL)		(Batch Size, 128 × 3)	
FC Layer.4 (joint)		(Batch Size, 128)	
FC Layer.5 (joint)		(Batch Size, 64)	
FC Layer.6 (joint)		(Batch Size, 2)	
**Batch Size**	**Learning Rate**	**Learning Rules**	**Dropout Rate**
100	0.001	RMSprop	0.5

**Table 8:** MDNN Architecture with Late Fusion.

Layers		Output Shape	
LSTM Layer (sensor)		(Batch Size, 128)	
Sensor Regression Output.1 ($X_{1}$, $Y_{1}$)		(Batch Size, 2)	
FC Layer.1 (WiFi)		(Batch Size, 128)	
Dropout Layer.1 (WiFi)		(Batch Size, 128)	
FC Layer.2 (WiFi)		(Batch Size, 128)	
Dropout Layer.2 (WiFi)		(Batch Size, 64)	
FC Layer.3 (WiFi)		(Batch Size, 32)	
WiFi Regression Output.2 ($X_{2}$, $Y_{2}$)		(Batch Size, 2)	
Fusion Network (input:$X_{1}$, $Y_{1}$, $X_{2}$, $Y_{2}$)		(Batch Size, 2 × 2)	
Regression Output.3 ($X_{3}$, $Y_{3}$)		(Batch Size, 2)	
**Batch Size**	**Learning Rate**	**Learning Rules**	**Dropout Rate**
100	0.001	RMSprop	0.5

**Table 9:** Comparative study of model performances under both scenarios.

Method	ScenarioA				ScenarioB			
	Min	Max	Mean	Std	Min	Max	Mean	Std
MM-Loc	**0.0331 m**	**30.1591 m**	1.5530 m	**1.7790 m**	**0.0031 m**	20.2881 m	**1.8859 m**	1.9679 m
P-MIMO	0.0866 m	30.3014 m	2.4021 m	2.9929 m	0.0059 m	21.3284 m	2.4363 m	2.0115 m
HDLM	0.0337 m	30.9279 m	1.9946 m	2.5993 m	**0.0031 m**	**18.4450 m**	2.1128 m	2.0198 m
GRU-CNN	0.0348 m	33.1648 m	**1.5446 m**	1.7886 m	0.0048 m	22.7855 m	2.1867 m	**1.8698 m**

## Data Availability

Not applicable.
